# miR-485-5p/NQO1 axis drives colorectal cancer progression by regulating apoptosis and aerobic glycolysis

**DOI:** 10.1186/s12935-025-03672-7

**Published:** 2025-02-12

**Authors:** Yixuan Wang, Houkun Zhou, Ying Liu, Xingyu Zhao, Shuhao Wang, Zhenhua Lin

**Affiliations:** 1 Central Laboratory, Yanbian University Hospital, No. 1327, Juzi-St., Yanji, Jilin Prov. 133000 China; 2 Department of Gastroenterology, Qingdao Municipal Hospital, Qingdao, 266071 China; 3 Key Laboratory of Pathobiology (Yanbian University), State Ethnic Affairs Commission, Yanji, 133000 China

**Keywords:** Colorectal cancer, NQO1, Proliferation, Migration, Glycolysis

## Abstract

**Background:**

Cancer cells undergo a metabolic shift termed the Warburg effect, transitioning from oxidative phosphorylation to aerobic glycolysis and promoting rapid tumor proliferation. Quinone oxidoreductase (NQO1), a cytosolic flavoprotein, is important for reprogramming cancer cell metabolism. Therefore, NQO1’s function in aerobic glycolysis and impact on colorectal cancer (CRC) development and progression was investigated.

**Methods:**

The clinical significance of NQO1 was evaluated by analyzing online databases and was substantiated in CRC specimens. NQO1’s influence on proliferation, epithelial-mesenchymal transition (EMT), metastasis, apoptosis, and glycolytic pathways in CRC cells was evaluated using in vitro and in vivo experiments. The molecular interactions between NQO1 and microRNA-485-5p (miR-485-5p) were ascertained via quantitative reverse transcription PCR and dual luciferase reporter assays. The molecular mechanisms underlying the miR-485-5p/NQO1 axis and its effects on progression of malignancy and aerobic glycolysis in CRC cell lines were investigated.

**Results:**

NQO1 promoted CRC cell proliferation and EMT, augmented their metastatic potential, and suppressed their apoptosis. The NQO1 overexpression-mediated enhancement of glycolytic activity is implicated in the increased proliferation, EMT, and metastatic abilities of, and reduced apoptosis in, CRC cells. Further, miR-485-5p may inhibit the proliferative and invasive traits of CRC cells by directly targeting the 3′ untranslated region of NQO1 mRNA.

**Conclusions:**

miR-485-5p/NQO1 signaling axis orchestrates aerobic glycolysis, thereby modulating CRC cell proliferation, metastasis, and apoptosis. Our study provides mechanistic perspectives regarding the role of NQO1 in CRC progression.

**Supplementary Information:**

The online version contains supplementary material available at 10.1186/s12935-025-03672-7.

## Background

Colorectal cancer (CRC) poses a significant threat to public health [1]. It is the second leading cause of oncologic mortality and accounts for 10.2% of all cancer diagnoses [2]. The predominant causes of death associated with CRC are distant metastasis and aggressive tumor proliferation [3].

Elevated glycolytic activity in neoplastic cells has been associated with malignant transformation and unfavorable prognosis in various cancers, including CRC [4]. Commonly, CRC cells exhibit a metabolic paradigm shift, transitioning from oxidative phosphorylation to aerobic glycolysis, an adaptation that confers augmented ATP production [5]. Further understanding the metabolic reprogramming of CRC cells from oxidative phosphorylation to aerobic glycolysis and identifying potent molecular markers and therapeutic targets, could pave the way for novel strategies to selectively inhibit aerobic glycolysis in CRC [6].

Quinone oxidoreductase (NQO1) causes the bioreductive transformation of quinones [7]. It plays an active role in the metabolism of various quinones, facilitating their bioactivation in vivo via electron transfer reactions [8]. Clinical evidence indicates that NQO1 expression is significantly elevated in numerous solid tumor cell types but remains minimally expressed in normal cells [9]. This distinctive overexpression and enzymatic activity renders NQO1 a prospective molecular therapeutic target for several malignancies [9]. However, the precise regulatory functions of NQO1 in CRC pathogenesis and progression remain unclear. Our previous study revealed that NQO1 was an essential regulator of altered glucose metabolism and contributed significantly to breast cancer progression [10]. Furthermore, the NQO1/p53 complex enhances the transcriptional activity of sterol regulatory element-binding protein 1, thereby driving the progression of hepatocellular carcinoma via altering lipid metabolism [11]. However, the intricate role of NQO1 in CRC progression and its impact on glucose metabolism warrant further investigation.

The microRNA-485-5p (miR-485-5p) has emerged as a significant regulatory element deeply involved in the etiology of various cancers, including CRC [12, 13]. Regulation of miR-485-5p expression by CircRUNX1 has been shown to promote the proliferation and metastatic ability of, and glutamine metabolism in, CRC cells while concurrently inhibiting apoptosis via the upregulation of SLC38A1 [14]. Moreover, miR-485-5p is a pivotal factor in CRC pathogenesis. Chai et al. reported that miR-485-5p was underexpressed in CRC tissue samples and cell lines, acting as a tumor-suppressive miRNA that impeded CRC cell growth and metastatic activity via attenuating the O-GlcNAcylation of Bmi-1 [15]. However, the specific mechanisms by which miR-485-5p influences aerobic glycolytic pathways in CRC progression remain unclear.

In the present study, we hypothesized that the miR-485-5p–NQO1 axis augmented aerobic glycolysis, thereby accelerating the growth and metastatic potential of CRC. The investigation of this hypothesis has elucidated the role of NQO1 in CRC progression, potentially yielding novel therapeutic targets and biomarkers for CRC diagnosis.

## Methods

### Ethical statement and patients

A cohort comprising 93 CRC specimens and 85 matched non-neoplastic colorectal tissues, accompanied by their respective clinicopathological metrics, was procured utilizing tissue microarray technology, acquired from Shanghai Outdo Biotech Co. Ltd. (Shanghai, China). Each tissue sample originated from accredited partner hospitals. The inclusion criteria for individuals with CRC required verifiable pathological diagnosis, acquisition of signed informed consent after a comprehensive patient information dialogue, and the completion of radical tumor excision procedures. The staging of clinicopathological data conformed to the staging system promulgated by the American Joint Committee on Cancer (AJCC).

**Fig. 1:**
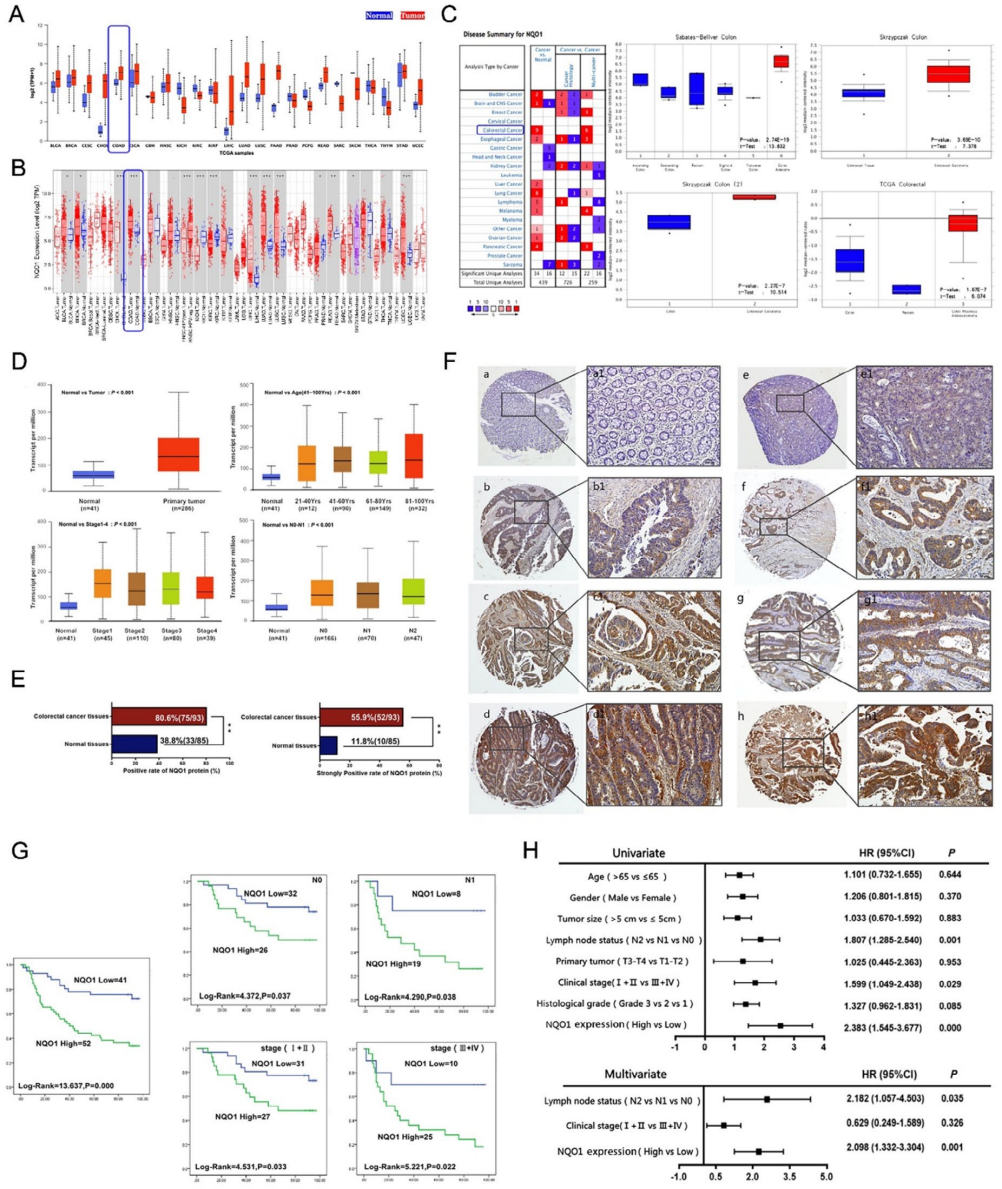
Upregulation of NQO1 predicted poor prognosis in patients with CRC. (**A-B**) UALCAN and TIMER database showed *NQO1* mRNA expression in normal and CRC tissues. (**C-D**) Oncomine and UALCAN database showed *NQO1* mRNA expression in normal and CRC tissues. (**E**) IHC staining showed the positive and strong positive rate of NQO1 protein expression in adjacent non-tumor (*n* = 85) and CRC (*n* = 93) tissues. (**F**) IHC staining images of the tissue microarray from CRC patients. (a-d) NQO1 protein expression in adjacent non-tumor (a-a1: negative) and CRC tissues (b-b1: weak positive; c-c1: positive; d-d1: strong positive); IHC images of NQO1 expression in Clinical stage I+II (e-e1) and III+IV. (f-f1); IHC images of NQO1 expression in CRC without lymph node metastasis (N0: g-g1) and tumors with lymph node metastasis (Nx: h-h1). Original magnification: 40× and 400×. (**G**) Kaplan–Meier survival curves showed the significance of NQO1 overexpression in CRC patients. (a) OS rates of patients with high (*n* = 52) and low (*n* = 41) NQO1 expression. (b–c) High NQO1 expression predicted the poor prognosis of patients without lymph node metastasis (*n* = 58) and with lymph node metastasis (*n* = 27). (d-e) High NQO1 expression predicted the poor prognosis of patients in Clinical stage I I+II (*n* = 58) and Clinical stage I+II (*n* = 35). (**H**) Forest plots of univariate and multivariate analysis of the clinicopathological features in 93 patients with CRC. *P < 0.05, **P < 0.01

**Fig. 2:**
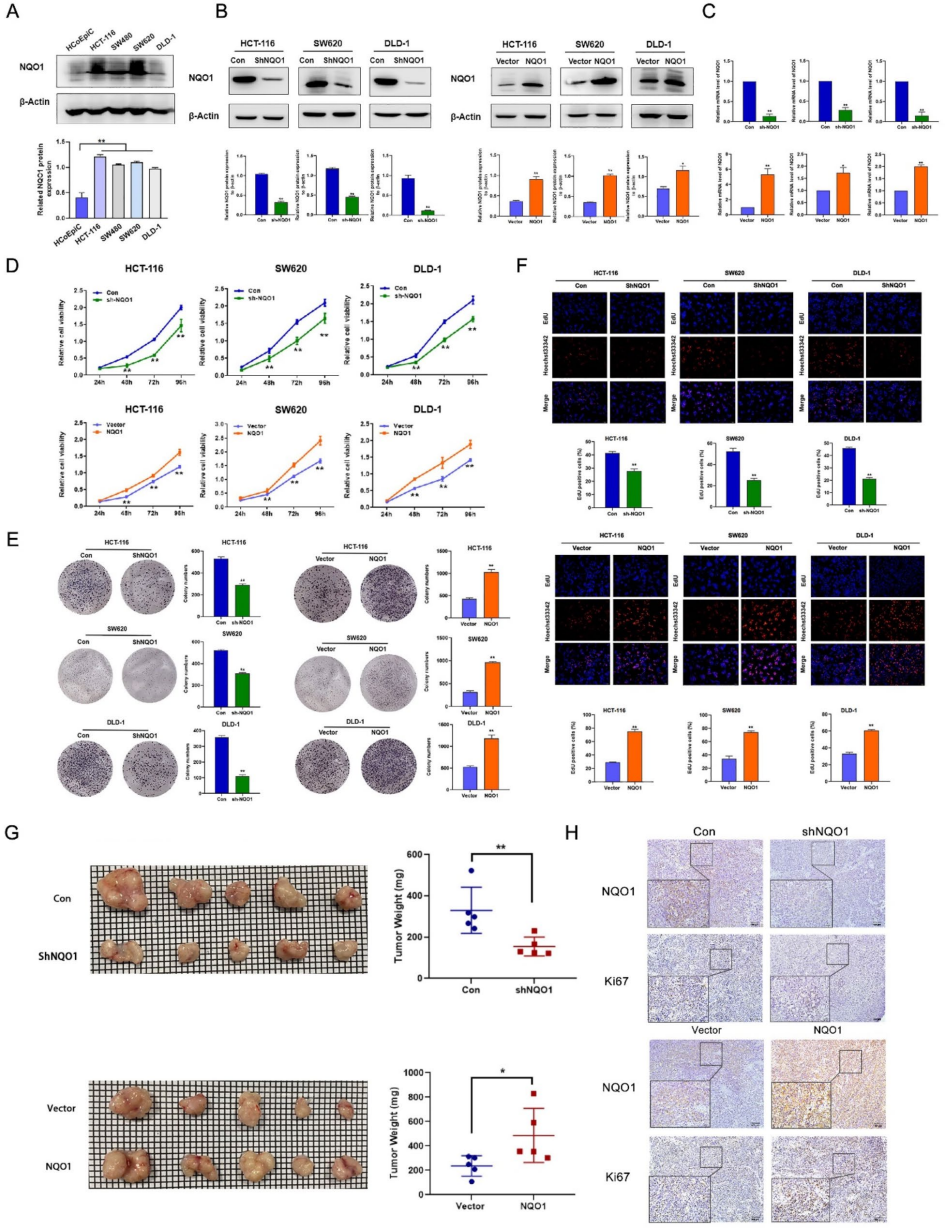
NQO1 promoted CRC cell proliferation in vitro and accelerated tumor growth in vivo. (**A**) Western Blot assay was used to detect the expression of NQO1 protein in normal colon epithelial cells and four CRC cell lines. (**B**) Western Blot assay was used to detect the expression of NQO1 protein in HCT-116, SW620 and DLD-1 after differential expression of NQO1. (**C**) The expression of NQO1 mRNA in HCT-116, SW620 and DLD-1 stably transfected cells was verified by qRT-PCR. (**D**) MTT assay was used to detect the effect of NQO1 differential expression on the activity of HCT-116, SW620 and DLD-1 cells. (**E**) Colony formation assay was used to detect the effect of NQO1 differential expression on the colony forming ability of HCT-116, SW620 and DLD-1 cells. (**F**) EdU assay were used to detect the effect of NQO1 differential expression on the proliferation ability of HCT-116, SW620 and DLD-1 cells. (**G**) The effect of NQO1 differential expression on the growth of xenograft tumors in nude mice (Upper: left is control group, and right is shNQO1 group. Lower: left is vector group, and right is NQO1 overexpression group) and the xenograft tumor weights were shown (*n* = 5 per group). Each small grid in the background represents a size of 1 mm × 1 mm. (**H**) IHC staining was used to detect the expression of NQO1 and Ki67 in the xenograft tumor tissues after differential expression of NQO1. *P < 0.05, **P < 0.01

### Bioinformatic analysis

The analysis employed databases such as GEPIA, Oncomine, and TIMER to investigate discrepancies in *NQO1* mRNA expression between CRC tissue samples and adjacent non-cancerous tissues. For the exploration of differentially expressed proteins, databases like STRING and Genemania were utilized, facilitating the elucidation of protein functions and the mapping of protein-protein interaction networks. Information on NQO1 expression, EMT-related markers, and metabolic enzymes was sourced from the GEPIA database, the starBase v3.0 portal, and Genemania. Gene Ontology (GO) enrichment analyses were conducted to determine the potential roles of NQO1 in cellular processes. Moreover, potential microRNA (miRNA) targets of NQO1 were forecasted and scrutinized with the assistance of four publicly available prediction platforms: TargetScan, miRDB, miRanda, and ENCORI.

### Cell culture and generation of stable cell lines

Colorectal cancer cell lines, namely SW480, SW620, HCT-116, and DLD-1, alongside the normal colonic epithelial cell line HCoEpic, were acquired from the American Type Culture Collection (ATCC) and subsequently propagated. Cultivation of these cells was conducted using RPMI 1640 Medium (supplied by Gibco Laboratories, Grand Island, NY), enriched with 10% fetal bovine serum (FBS) (also sourced from Gibco), 100 U/mL of penicillin, and 100 µg/mL of streptomycin. This was performed at a controlled temperature of 37 °C within an atmosphere containing 5% CO2. For the generation of stable cell lines, lentiviral vectors were utilized to modulate NQO1 expression, either enhancing or silencing its activity (provided by Genechem, Shanghai, China). Post-transfection, cells were subjected to selection via 2 µg/ml puromycin over two weeks. MicroRNA agents, including the miR-484-5p mimic, a nonspecific miRNA control, a miR-485-5p inhibitor, and a nonspecific miRNA inhibitor control, were obtained from RIBOBIO (China). In adherence to the supplier’s protocol, transfections of miRNA were executed employing Lipofectamine 3000 reagent within Opti-MEM reduced serum medium (courtesy of Invitrogen, USA).

**Fig. 3:**
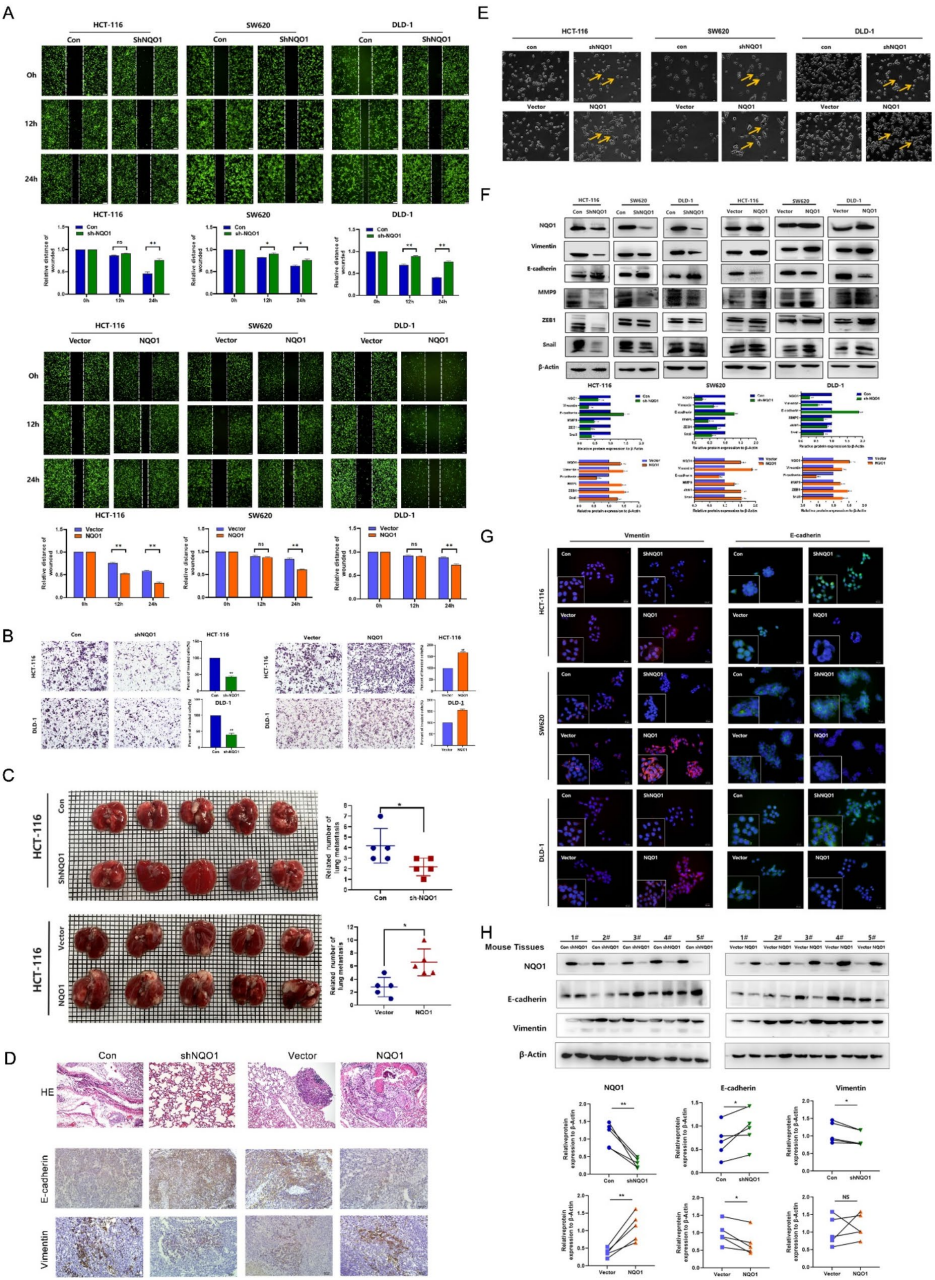
NQO1 promoted the metastasis of CRC cells via EMT process in *vitro* and in *vivo*. (**A**) Wound healing assay displays the cell migration after 24 h in CRC cells after differential expression of NQO1. (**B**) Transwell cell assay was used to detect the changes of migration ability of HCT-116 and DLD-1 cells after differential expression of NQO1. (**C**) Lung metastasis model was used to detect the effect of NQO1 differential expression on the metastatic ability of colorectal cancer cells in nude mice (*n* = 5 per group). Representative images showed the number of lung metastasis nodules, the size of the tumors. (**D**) HE staining of the lung tissues and IHC staining of E-cadherin and Vimentin in the four groups of xenograft tumor tissues. (**E**) Morphological changes of HCT-116 and DLD-1 cells after lentivirus transfection with differential expression of NQO1. (**F**) Western blot assay was used to detect the protein expression of EMT-related markers and transcription factors in CRC cells after differential expression of NQO1. (**G**) IF staining assay was used to detect the expression of epithelial marker E-cadherin and mesenchymal marker Vimentin in CRC cells after differential expression of NQO1 (blue: nuclear staining; green: E-cadherin staining; red: Vimentin staining). (**H**) Western blot assay detects the expression of EMT-related markers in the xenograft tumor tissues after differential expression of NQO1. **P < 0.01

**Fig. 4:**
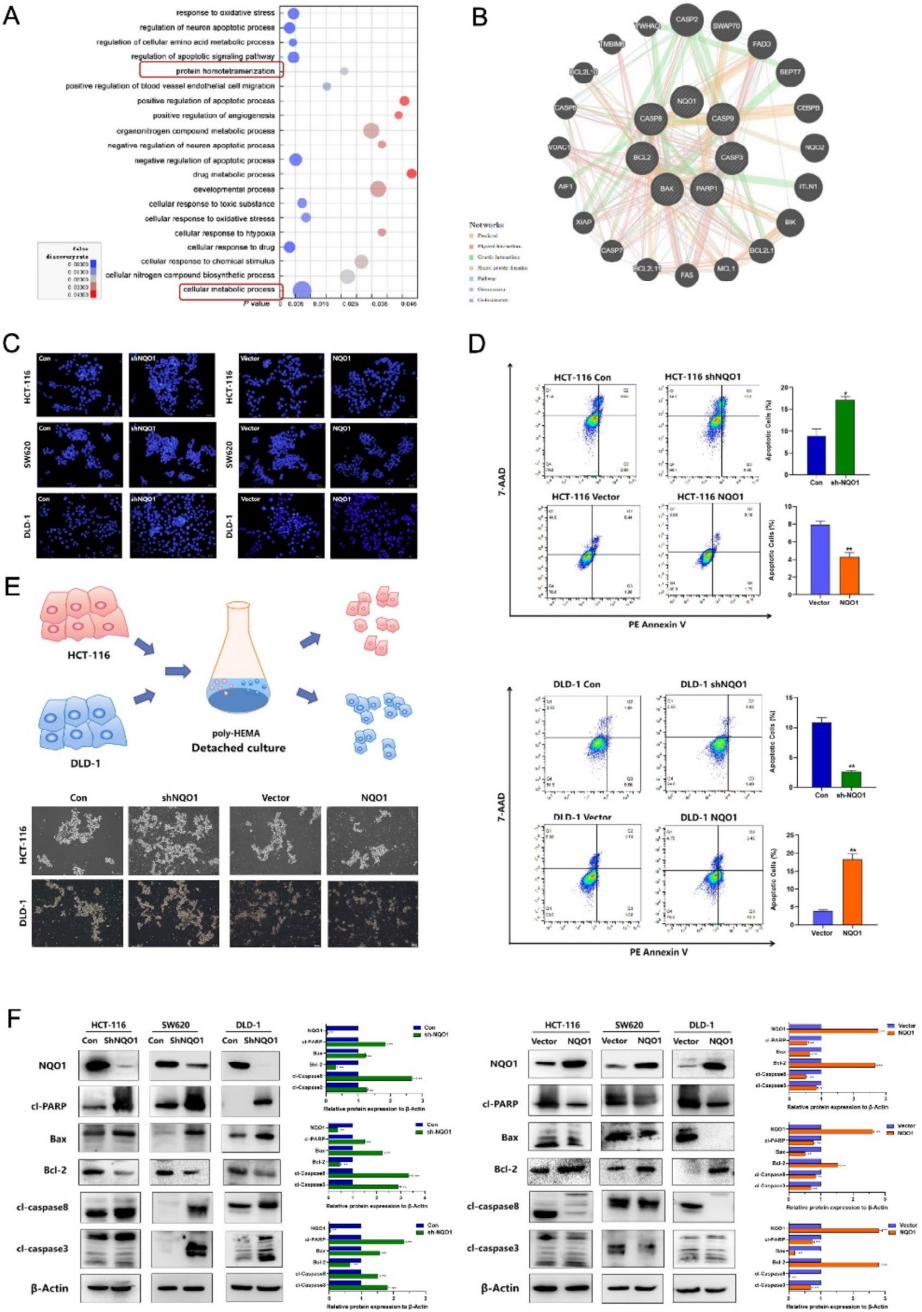
NQO1 Promotes anoikis resistance of CRC cells. (**A-B**) GO pathway analysis and GeneMANIA found that NQO1 may participate in biological processes and the correlation between NQO1 and apoptosis-related protein. (**C**) The effect of differential expression of NQO1 on apoptosis of CRC cells was detected by Hoechst 33,342 staining. (**D**) The effect of differential expression of NQO1 on apoptosis of CRC cells was detected by Annexin V-PE/7-AAD assay using flow cytometry. (**E**) Differential expression of NQO1 on HCT-116 and DLD-1 cells were cultured successfully and photographed under microscope. (**F**) Western blot determination of apoptosis markers protein expression in CRC cells after differential expression of NQO1

### Quantitative real-time polymerase chain reaction (qRT-PCR)

Total ribonucleic acid (RNA) was isolated utilizing TRIzol Reagent (Invitrogen, CA, USA), after which it was reverse transcribed into complementary DNA (cDNA) utilizing the Superscript First-strand Synthesis System kit (Invitrogen), adhering to the protocols provided by the manufacturer. qRT-PCR assays were performed in triplicate employing SYBR Premix Ex Taq II (TaKaRa) on a Roche LightCycler^®^ 96 real-time PCR system. The relative gene expression was normalized to the expression of GAPDH and calculated using the 2 ^−ΔΔCt^ method. Primer sequences specific to the genes of interest for the qRT-PCR are enumerated in Supplementary Table 1.

### Immunohistochemical staining analysis

Tissue specimens, encompassing clinical samples and xenograft tumors, underwent initial fixation in formalin, followed by immunohistochemical (IHC) staining as delineated in previous work [16]. The immunostaining intensity was independently evaluated and scored by two pathologists. A composite staining score was then computed, integrating both the staining intensity and the proportion of immunopositive cells. In the case of mouse lung tissues, they were fixed with 4% paraformaldehyde (PFA) for 12 h, subjected to a sequential dehydration process, clarified, and then embedded in paraffin. Subsequent to sectioning, the tissues were stained with hematoxylin and eosin (H&E), after which they were coverslipped and examined.

### In vitro cell behavior assays (cell proliferation and migration assay)

Cellular proliferation was evaluated using a suite of assays including MTT cell viability, colony formation, and 5-ethynyl-2’-deoxyuridine (EdU) incorporation tests. For the MTT assay, cells were seeded into 96-well plates and treated with 5 mg/mL of MTT reagent. After four hours of incubation, the medium was removed and the cells were washed with phosphate-buffered saline (PBS). Dimethyl sulfoxide (DMSO) was then added to dissolve the formazan crystals formed, and the absorbance was measured at 570 nm using a microplate reader. In the colony formation assays, cells were plated at a density of 1,000 per well in 6-well plates and cultivated for a fortnight. Colonies that emerged were fixed with 4% paraformaldehyde, stained with Giemsa, and imaged using an Olympus BX53 microscope. The EdU assay was executed following the instructions provided with the RiboBio EdU kit.

Cell migration was assessed by wound healing and transwell assays. The wound healing assay involved creating a simulated wound in a cell monolayer with a 200 µL pipette tip. Cells were imaged immediately and after 24 h using a Nikon Eclipse TE300 microscope. Transwell migration assays involved seeding cells into upper chambers with serum-free medium, while the bottom chambers contained 20% FBS-enriched medium. Following migration, cells were fixed with 4% paraformaldehyde and stained with crystal violet. A BX53 Olympus microscope captured the images, and migrated cells were enumerated in three to five randomly selected fields.

### Immunofluorescence (IF) staining

Samples were affixed to coverslips using a 4% paraformaldehyde solution and subjected to a low-temperature incubation at 4 °C with a primary antibody in a solution enriched with 3% bovine serum albumin (BSA). Subsequent to the initial incubation, the samples were incubated with secondary antibodies conjugated to Alexa Fluor 488 or Alexa Fluor 546. Cell nuclei were subsequently stained using 4’,6-diamidino-2-phenylindole (DAPI), employing the C1006 assay (Biotime, Shanghai, China) for assistance. Following staining, an anti-fade reagent was applied, and the coverslips were mounted. The cells were then examined and imaged using an Olympus BX53 microscope.

### Western blot analysis

Following extraction, proteins were denatured by heating at 100 degrees Celsius for five minutes. The denatured proteins were then separated via 8–10% gradient sodium dodecyl sulfate-polyacrylamide gel electrophoresis (SDS-PAGE). Subsequently, the separated proteins were transferred onto polyvinylidene difluoride (PVDF) membranes supplied by Millipore (Billerica, MA). These membranes were then blocked with a 5% skim milk solution and incubated at 4 °C overnight with meticulously selected primary antibodies. Prior to the primary antibody incubation, the membrane was treated with the appropriate secondary antibody for one hour at ambient temperature. Protein bands were visualized using state-of-the-art enhanced chemiluminescence (ECL) reagents, with the emitted luminescence captured and quantitatively analyzed using Bio-Rad’s Image Lab software (Hercules, CA).

### Cell apoptosis assay

For the identification of apoptotic cells, the Annexin V-FITC Apoptosis Detection Kit by BioVision (Palo Alto, CA) was utilized. The protocol began with the harvesting of cells followed by a double rinse in chilled PBS. Thereafter, the cells were resuspended in a binding buffer, attaining a concentration of 300,000 cells per tube. Subsequently, each cell suspension was augmented with 10 µL of propidium iodide (PI) and 5 µL of Annexin V-FITC. The cells were then incubated in the dark to prevent photobleaching. Following incubation, cells were analyzed for apoptotic markers using a DxFLEX flow cytometer from Beckman Coulter (Indianapolis, IN, USA), employing flow cytometry techniques.

### Luciferase reporter assay

Cells were seeded at a concentration of 200,000 cells for each well into 24-well culture plates designed for tissue culturing. Each subsequent experimental procedure was rigorously adhered to as prescribed by the Lipofectamine™ 3000 protocol (Invitrogen, USA). Throughout the co-transfection stage, cells were administered either wild-type or mutant *NQO1* 3’UTR luciferase reporter constructs in conjunction with miR-485-5p or a nonspecific control miRNA. Twenty-four hours post-transfection, the cells were collected and lysed utilizing a lysis buffer provided by Promega. Quantification of luciferase activity was conducted utilizing the Dual-Luciferase^®^ Reporter Assay System by Promega.

### Seahorse assay

The Seahorse XF96 Extracellular Flux Analyzer, developed by Seahorse Biosciences, was employed to ascertain the rates of extracellular acidification (ECAR) and oxygen consumption (OCR) within the cultured cells. Each well of the XF96 culture plate was seeded with 12,000 cells for the experimental analysis. The cells were equilibrated in a custom assay medium at a controlled temperature of 37 °C for one hour prior to the commencement of baseline measurements. Following the establishment of initial metrics, an array of chemical agents, pre-diluted in the assay medium, were sequentially administered to each well. This approach facilitated the real-time monitoring of changes in both ECAR and OCR. ECAR was quantified in milli pH units per minute (mpH/min) and normalized to the protein content measured in each well. OCR readings were likewise recorded in picomoles per minute (pmol/min) and adjusted based on the corresponding protein concentrations. To culminate the procedure, cells were rapidly detached using trypsin, ensuring that the rate measurements obtained from each well were duly corrected for variations in protein density.

### Glucose uptake, lactate, and ATP production assay

To quantify the glucose consumption, lactate secretion, and ATP levels in CRC cells, three distinct assays were employed: the Glucose Uptake Colorimetric Assay Kit from Rongsheng Biotech Co., Ltd., and both the Lactate and ATP Assay Kits from Nanjing Jiancheng Bioengineering Institute. The execution of each assay followed the precise guidelines provided by each kit’s manufacturer.

### Nude mice xenograft

Nude BALB/c mice, 5 weeks of age, were inoculated subcutaneously with 3 × 10^6^ HCT-116 cells on either the left or right flank to establish a tumor model. To induce lung metastasis, 1 × 10^6^ HCT-116 cells were intravenously injected via the tail vein of each mouse. Following a period of 8 weeks, the animals were humanely euthanized. The harvested xenografts were then processed; one segment was fixed in 10% buffered formalin for histopathological examination, and another was immediately cryopreserved in liquid nitrogen for further investigation. All animal-related procedures were conducted in strict compliance with the guidelines set forth by the Animal Ethics Committee of Yanbian University, China.

**Fig. 5:**
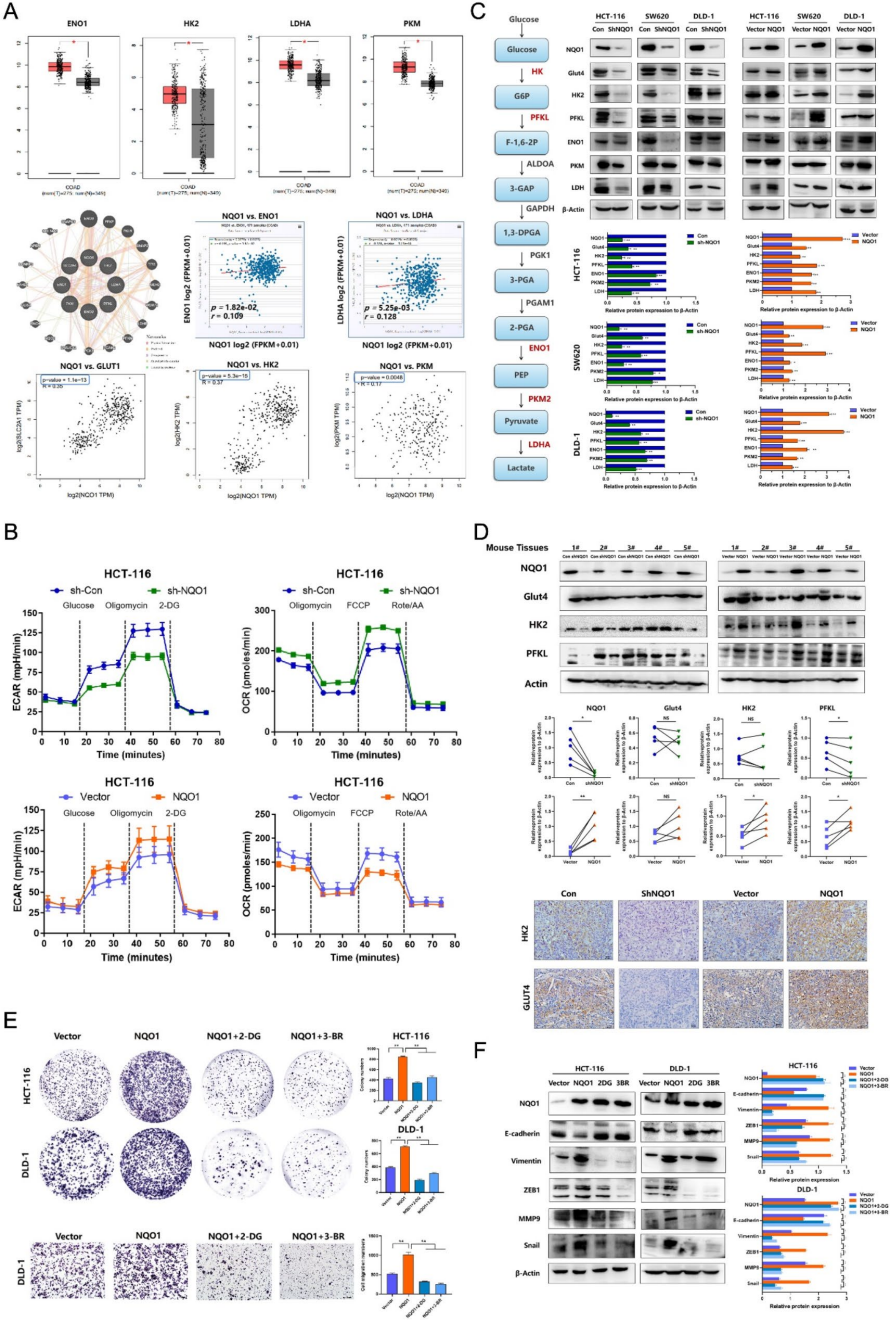
NQO1 regulates the malignant progression of CRC cells through aerobic glycolysis. (**A**) GEPIA database showed that the mRNA expression of glycolytic markers in CRC tissue was significantly up-regulated; GeneMANIA, GEPIA and StarBase v3.0 databases showed the correlation between NQO1 mRNA and glycolysis markers mRNA; (**B**) Seahorse mitochondrial stress test quantitating OCR and ECAR in NQO1 differential expression groups of HCT-116 cells. (**C**) Western blot examined the expression of the glucose transporters and metabolic enzymes in CRC cells after differential expression of NQO1. (**D**) Western blot and IHC examined the expression of the glycolysis markers in CRC cells after differential expression of NQO1. (**E**) Glycolysis inhibitors 2-DG and 3-BrPA reverse NQO1 to promote the proliferation and migration of CRC cells by Colony formation assay and transwell assay. (**F**) Western blot showed that 2-DG and 3-BrPA reversed the down-regulation of E-cadherin expression and up-regulation of Vimentin and MMP9, ZEB1, Snail expression caused by the overexpression of NQO1. *P < 0.05, * * *P* < 0.01

**Fig. 6:**
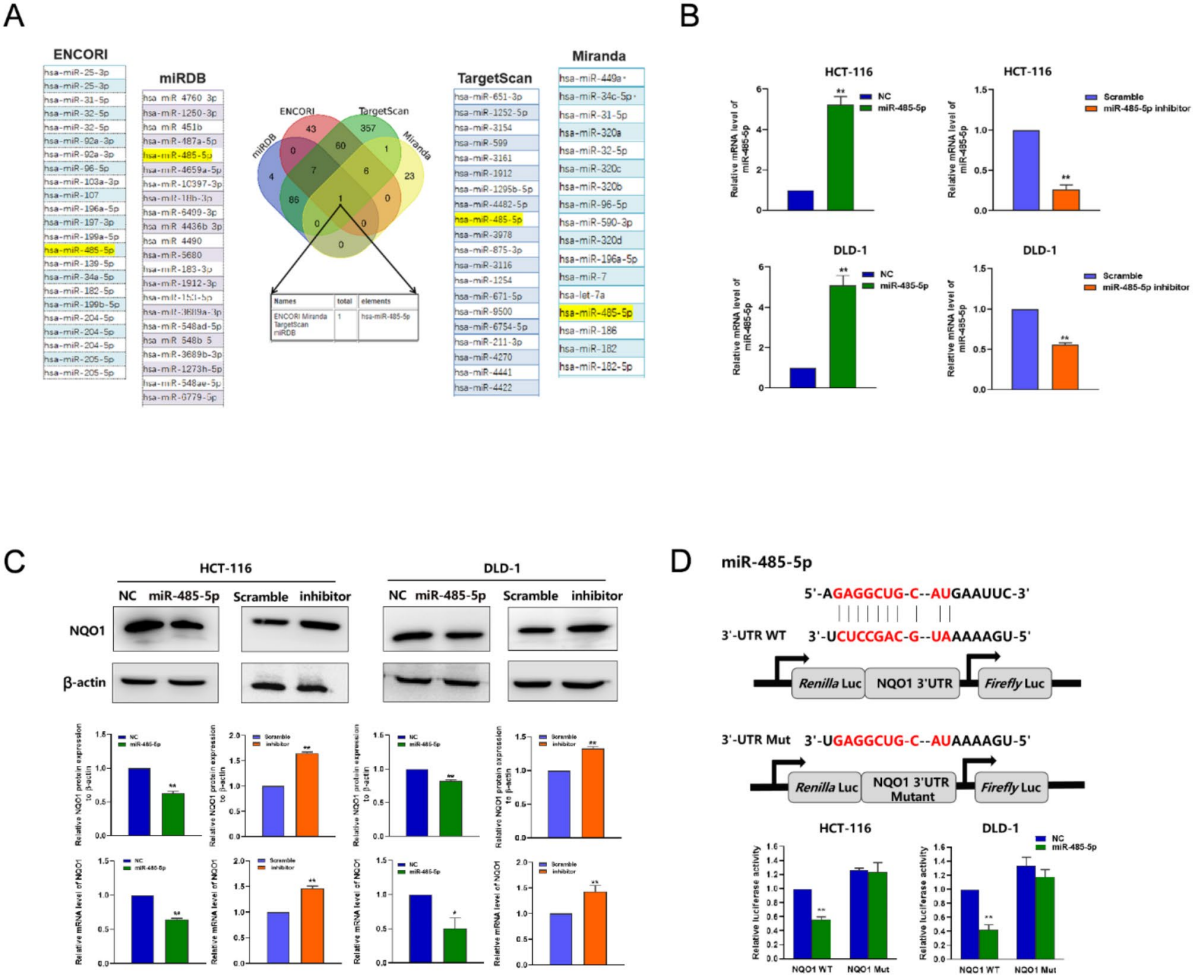
miR-485-5p directly targeted *NQO1*. (**A**) Venn diagrams showed the overlap from four independent databases and found possible candidate upstream regulators of *NQO1*. (**B**) qRT-PCR showed the miR-485-5p mRNA expression transfection of miR-485-5p mimic and miR-485-5p inhibitor. (**C**) qRT-PCR and Western blot showed the expression levels of *NQO1* mRNA and protein after transfection of miR-485-5p mimic and miR-485-5p inhibitor. (**D**) The binding sites of 3’-UTR and miR-485-5p. Verification of the effect of miR-485-5p targeting *NQO1* by Luciferase reporter assay. *P < 0.05, * * *P* < 0.01

### Statistical methods

Statistical analysis of the data was performed using the SPSS software (Version 25.0, SPSS Inc, Chicago, IL, USA). In parallel, GraphPad Prism (Version 8.0) was utilized for the generation of graphical representations of the data. The relationships between NQO1 expression and diverse clinicopathological characteristics were investigated by employing either the Chi-square (χ2) test or Fisher’s exact test, depending on which was more suitable for the analysis at hand. Overall survival (OS) was assessed utilizing Cox’s proportional hazards model, in concert with the Kaplan-Meier estimator and the log-rank test, all within the SPSS software framework. For determining statistical significance in comparative analyses, both the Student’s t-test and one-way ANOVA were employed. Results are articulated as mean ± standard deviation (SD), based on a minimum of three independent experiments. Statistical significance was designated by *P < 0.05 and **P < 0.01 to distinguish the levels of confidence.

## Results

### Elevated NQO1 expression associates with advanced clinicopathological profiles and poor prognostic predictors in patients with CRC

Using several bioinformatics tools, including UALCAN, TIMER, and the GEPIA databases, we examined the expression profile of NQO1 in CRC. Comparative analyses revealed a pronounced elevation in *NQO1* transcript levels in CRC tissues relative to those in adjacent normal colonic tissues (Fig. 1A-C). Further investigation using the UALCAN database to scrutinize *NQO1* mRNA expression in conjunction with clinicopathological variables across 41 colorectal and 286 CRC specimens demonstrated that increased NQO1 expression significantly correlated with age, clinical stage, and lymph node involvement in patients with CRC (Fig. 1D, *P* < 0.01).

Subsequent immunohistochemical (IHC) analysis of 93 paraffin-embedded CRC samples and 85 non-cancerous tissues confirmed that the NQO1 protein expression was conspicuously elevated in CRC tissues (Fig. 1E, *P* < 0.01), with increments being observed parallel to clinical stage progression and lymph node metastasis (Fig. 1F, *P* < 0.01). Correlation analysis revealed that NQO1 overexpression was significantly associated with clinical stage and lymph node metastasis (*P* < 0.05) (Table 1). However, no such association was found with sex, tumor dimensions, primary tumor site, or histological grade, corroborating the IHC findings.

**Table 1:** Relationship between NQO1 protein overexpression and the clinicopathological parameters of CRC

Variables	No. of case (*n*)	NQO1	χ^2^	*P* value
		Strong positive rate (%)		
**Gender**				
Male	44	27 (61.4%)	1.006	0.316
Female	49	25 (51.0%)		
**Age (years)**				
>65	49	27 (55.1%)	0.028	0.868
≤65	44	25 (56.8%)		
**Tumor size**				
≤5 cm	62	36 (58.1%)	0.349	0.555
>5 cm	31	16 (51.6%)		
**Histological grade**				
Grade-1	20	11 (55.0%)	1.325	0.515
Grade-2	57	30 (52.6%)		
Grade-3	16	11 (68.8%)		
**Lymph node status**				
N0	58	26 (44.8%)	8.419	0.015*
N1	27	19 (70.4%)		
N2	8	7(87.5%)		
**Primary tumor status**				
T1-T2	6	5 (83.3%)	1.956	0.162
T3-T4	87	47 (54.0%)		
**Clinical stage**				
I+II	58	27 (46.6%)	5.832	0.019*
III+IV	35	25 (71.4%)		

* *P*<0.05

Additionally, survival analysis using Kaplan-Meier curves showed that patients with CRC who had high levels of NQO1 expression experienced significantly reduced survival times compared with those with lower expression levels (log-rank = 13.637, *P* = 0.000) (Fig. 1G). Single variable analysis reinforced this result, revealing a significant correlation between overall survival in patients with CRC and factors such as lymph node metastasis (*P* = 0.001), clinical stage of the disease (*P* = 0.029), and NQO1 expression status (*P* = 0.000), thus supporting the potential of NQO1 as a strong prognostic indicator of CRC. Furthermore, the Cox proportional hazards model highlighted the expression of the NQO1 protein (*P* = 0.001) and lymph node metastasis (*P* = 0.035) as significant independent predictors of survival (Fig. 1H).

**Fig. 7:**
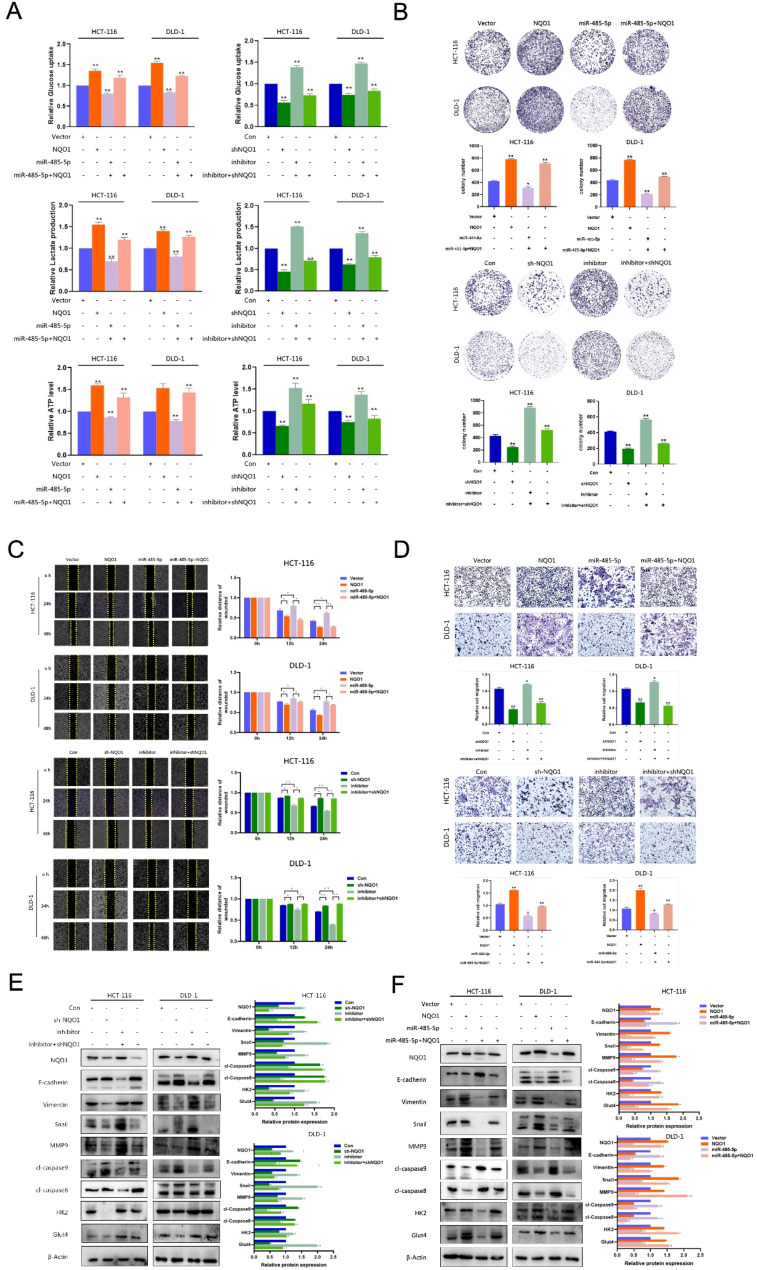
The miR-485-5p/NQO1 regulated aerobic glycolysis in CRC. (**A**) Effects of miR-485-5p/NQO1 axis on glucose uptake, lactate and ATP production in miR-485-5p inhibitor or miR-485-5p mimic and miR-485-5p inhibitor + sh-NQO1 or miR-485-5p + NQO1 cells. (**B**) Effects of miR-485-5p/NQO1 axis on the proliferation of miR-485-5p inhibitor or miR-485-5p mimic and miR-485-5p inhibitor + sh-NQO1 or miR-485-5p + NQO1 cells was detected by Colony formation assay. (**C**) Effect of miR-485-5p/NQO1 axis on the migration ability of miR-485-5p inhibitor or miR-485-5p mimic and miR-485-5p inhibitor + sh-NQO1 or miR-485-5p + NQO1 cells was detected by wound healing assay. (**D**) Effect of miR-485-5p/NQO1 axis on the migration ability of miR-485-5p inhibitor or miR-485-5p mimic and miR-485-5p inhibitor + sh-NQO1 or miR-485-5p + NQO1 cells was detected by transwell assays. (**E**) Effect of miR-485-5p/NQO1 axis on EMT, apoptosis and glycolysis related marker proteins in miR-485-5p inhibitor or miR-485-5p mimic and miR-485-5p inhibitor + sh-NQO1 or miR-485-5p + NQO1 cells was detected by Western blot. *P < 0.05, * * *P* < 0.01

**Fig. 8:**
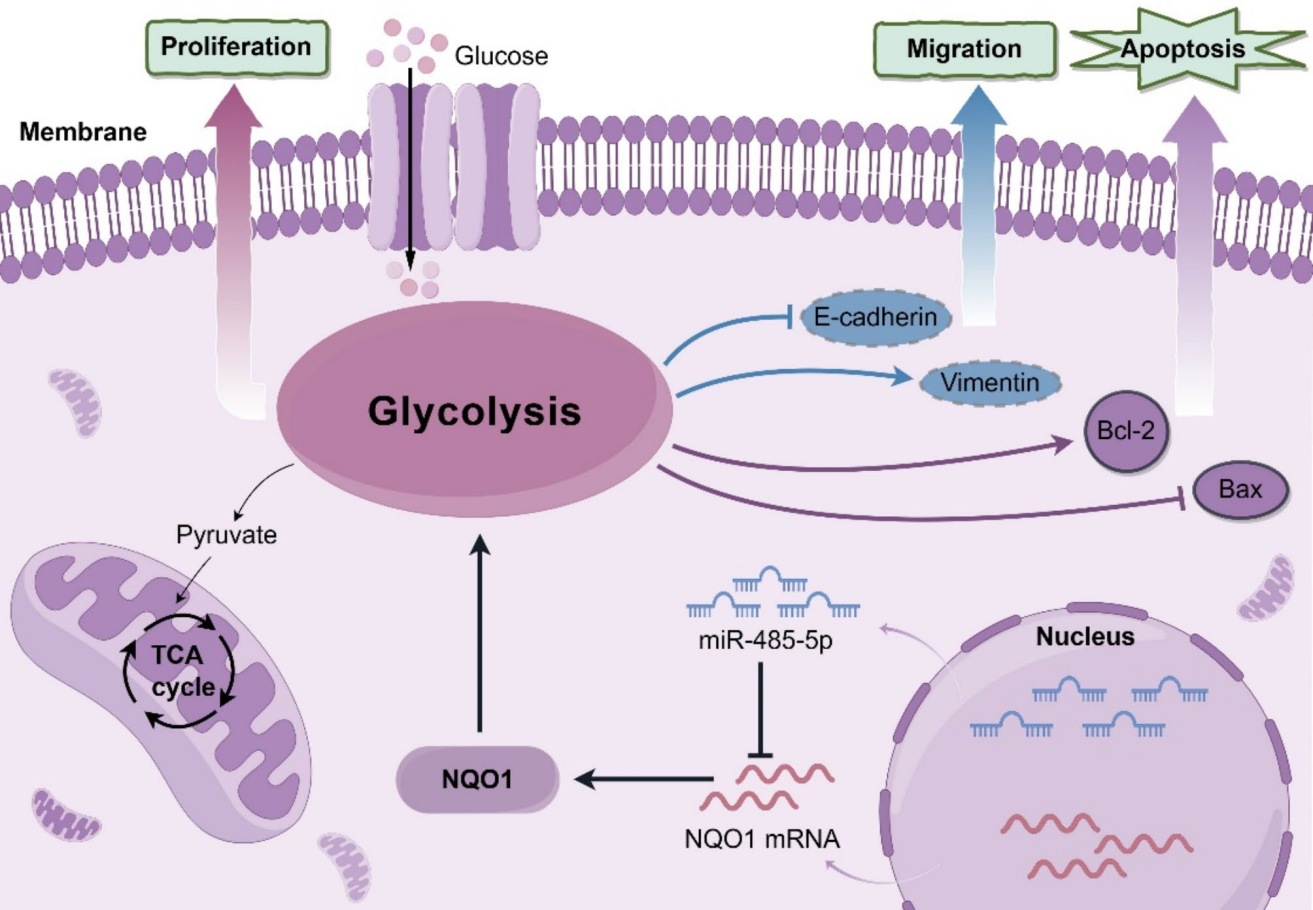
Schematic illustration of the mechanism of miR-485-5p/NQO1 mediated aerobic glycolysis in the regulating of progression in CRC. By Figdraw

### NQO1 promotes CRC cell proliferation both in vitro and in vivo

Initially, NQO1 protein expression was determined in human primary colonic epithelial cells (HCoEpiCs) and four CRC cell lines (HCT-116, SW480, SW620, and DLD-1) using western blot analysis. The results indicated a significant upregulation of NQO1 in CRC cell lines compared to HCoEpiCs (Fig. 2A). Subsequently, HCT-116, SW620, and DLD-1 cells were subjected to either upregulation or downregulation of NQO1 expression via lentiviral transduction. The efficiency of these manipulations was confirmed via western blotting and quantitative reverse transcription polymerase chain reaction (Fig. 2B, C).

To evaluate the influence of NQO1 on the proliferative capacity of CRC cells, a series of assays including MTT, colony formation, and EdU incorporation assays were performed. The MTT and colony formation assays consistently showed that NQO1 knockdown significantly reduced the proliferation and clonogenic potential of CRC cells. Conversely, NQO1 overexpression enhanced these cellular characteristics (*P* < 0.01) (Fig. 2D, E). Additionally, EdU assays demonstrated that NQO1 suppression significantly impeded DNA synthesis in CRC cells, whereas its overexpression significantly increased DNA synthesis (*P* < 0.01) (Fig. 2F).

In our in vivo tumorigenicity assays, xenograft models were generated using HCT-116 cells with stably altered NQO1 expression. Tumor growth monitoring indicated that relative to the control group, xenografts from the NQO1-silenced group showed significantly reduced tumor volume and weight (*P* < 0.01). In contrast, xenografts from the NQO1-overexpressing group exhibited considerable increases in both tumor volume and weight (Fig. 2G). Furthermore, IHC staining for Ki67, a marker of cell proliferation, showed decreased and increased Ki67 expression levels in the NQO1 knockdown and overexpressing groups, respectively, compared to their respective controls (Fig. 2H). Collectively, these findings support the oncogenic role of NQO1 in CRC.

### NQO1 promotes the metastasis and EMT of CRC cells both in vitro and in vivo

IHC studies revealed a significant correlation between elevated NQO1 levels and the presence of lymph node metastases, suggesting that NQO1 was involved in CRC dissemination. Functional assays, including wound healing and Transwell assays, demonstrated a significant reduction in the migratory capabilities of CRC cells upon NQO1 knockdown. Conversely, increased NQO1 levels were associated with enhanced cell migration (*P* < 0.05) (Fig. 3A, B). In a lung metastasis model, the downregulation of NQO1 expression substantially impeded the CRC cell capacity to metastasize to the lungs, as confirmed by hematoxylin and eosin staining of lung tissue sections. Quantitative analysis of these sections revealed a significant decrease in the number of metastatic lung nodules upon NQO1 silencing, whereas an increase in the number of such nodules was observed after NQO1 overexpression (*P* < 0.05) (Fig. 3C, D).

EMT has a crucial role in tumor invasiveness and metastasis; hence, we investigated the NQO1 influence on this process. Morphological observations indicated that cells with NQO1 overexpression adopted a spindle-like mesenchymal morphology, whereas NQO1-silenced cells exhibited a cobblestone-like epithelial appearance (Fig. 3E). Subsequent western blotting and immunofluorescence assays showed that NQO1 silencing was associated with the upregulation and downregulation of epithelial and mesenchymal markers, respectively (*P* < 0.01). The converse was true for cells with NQO1 overexpression (Fig. 3F, G). Molecular alterations were corroborated by western blotting and IHC analyses in tumor tissue models, reinforcing the findings. Collectively, these results provide compelling evidence that NQO1 facilitates CRC metastasis and EMT both in vivo and in vitro (Fig. 3H).

### NQO1 promotes anoikis resistance of CRC cells

Gene ontology enrichment analysis suggested a role for NQO1 in apoptosis (Fig. 4A). Subsequent interrogation using GeneMANIA and protein-protein interaction networks revealed potential interactions between NQO1 and apoptosis-associated proteins (Fig. 4B). Hoechst staining revealed a significant increase in the number of bright blue apoptotic bodies in CRC cells following NQO1 knockdown, indicating enhanced apoptotic activity. Conversely, NQO1 overexpression suppressed the occurrence of these apoptotic features (Fig. 4C). Anoikis is a distinct form of programmed cell death that occurs when a cell loses contact with neighboring cells or the extracellular matrix (ECM). For tumor cells to acquire the ability to metastasize, they must develop resistance to anoikis, enabling them to survive without adhering to the ECM [17]. This particular form of apoptosis plays a crucial role in preventing the progression of aggressive and metastatic tumors. To investigate whether NQO1 is involved in the regulation of anoikis, HCT-116 and DLD-1 cells were cultured on poly-2-hydroxyethyl methacrylate-coated plates to prevent attachment, simulating an anoikis environment, and cellular morphology was evaluated microscopically (Fig. 4D). Flow cytometry analysis demonstrated an increased apoptosis rate in cells with NQO1 knockdown, whereas those with NQO1 overexpression exhibited a decreased apoptosis rate (*P* < 0.01) (Fig. 4E).

Western blotting was used to quantify levels of proteins associated with apoptotic processes. These analyses revealed that NQO1 upregulation increased the expression of the anti-apoptotic protein BCL2 and concurrently decreased the expression of pro-apoptotic proteins, including BAX, cleaved PARP, cleaved Caspase-8, and cleaved Caspase-3 (Fig. 4F). In contrast, NQO1 silencing resulted in the anticipated inverse effect. Collectively, these findings support the hypothesis that NQO1 contributes to anoikis resistance in CRC cells.

### NQO1 promotes the malignant progression of CRC cells via aerobic glycolysis

Recent studies have shown that CRC cells rely on aerobic glycolysis to synthesize ATP, thereby providing the energy necessary for proliferation and metastasis [18]. Functional enrichment analysis, protein-protein interaction studies, and data from the GEPIA database collectively indicated a significant positive correlation between NQO1 expression and mRNA levels of pivotal glycolytic enzymes, suggesting a regulatory role for NQO1 in aerobic glycolysis in CRC (Fig. 5A). The bioenergetic profiles of CRC cells, as assessed using the Seahorse XFe96 Analyzer, demonstrated that NQO1 knockdown reduced the glycolytic ability and enhanced the oxidative phosphorylation ability. Conversely, NQO1 upregulation yielded the opposite effect (Fig. 5B). Western blot analyses further confirmed these findings, showing that lower NQO1 expression diminished levels of the glucose transporter GLUT4 and various glycolytic enzymes (hexokinase II, phosphofructokinase-1, enzyme enolase-1, pyruvate kinase, and lactate dehydrogenase), whereas NQO1 overexpression augmented glycolytic activity (Fig. 5C). Additional western blot analyses revealed that hexokinase II and phosphofructokinase-1 protein levels decreased in tumor tissues from the NQO1 knockdown group and increased in those from the overexpression group; these results were substantiated via IHC staining (Fig. 5D). Collectively, these results suggest that NQO1 facilitates aerobic glycolysis in CRC both in vitro and in vivo.

Subsequent investigations focused on whether the regulatory effects of NQO1 on CRC cell proliferation and metastasis occurred via glycolysis. The glycolytic inhibitors 2-deoxy-D-glucose and 3-bromopyruvate, at a concentration of 10 mM, significantly impeded the NQO1 ability to enhance CRC cell proliferation and migration. (Fig. 5E). Furthermore, these inhibitors effectively abrogated the NQO1-mediated upregulation of the mesenchymal markers Vimentin, Snail, MMP9, and ZEB1 and the downregulation of epithelial marker E-cadherin (*P* < 0.05) (Fig. 5F). Collectively, these findings suggest that NQO1 modulates CRC cell proliferation and metastasis via mediating aerobic glycolysis.

### *NQO1* identified as a direct target gene of mir-485-5p in CRC cells

To elucidate the complex regulatory mechanisms governing NQO1 expression, we used four miRNA target prediction algorithms: miRanda, ENCORI, miRDB, and TargetScan. This comprehensive approach identified miR-485-5p as a candidate regulatory miRNA, as illustrated in Fig. 6A. The 3′-untranslated region (UTR) of *NQO1* possesses a complementary binding site for miR-485-5p. Experimental interventions, including the enforced upregulation of miR-485-5p, reduced *NQO1* expression at both mRNA and protein levels in CRC cells. Conversely, inhibition of miR-485-5p increased NQO1 expression, as shown in Fig. 6B and C.

Further substantiation of the direct interaction between miR-485-5p and *NQO1* was achieved using a luciferase reporter assay. Plasmids harboring the wild-type or mutated *NQO1* 3′-UTR were transfected into HCT-116 and DLD-1 cell lines, concomitantly with miR-485-5p mimics or non-targeting control sequences. The introduction of miR-485-5p mimics significantly attenuated luciferase activity in vectors containing the unmodified *NQO1* 3′-UTR, indicating a targeted binding interaction (*P* < 0.05), as depicted in Fig. 6D. This inhibitory effect was not observed in vectors with mutations in the miR-485-5p-binding sites. Collectively, these results demonstrate that miR-485-5p selectively targets *NQO1* in CRC cells.

### The miR-485-5p/NQO1 axis promotes aerobic glycolysis in CRC cells

To elucidate the effects of miR-485-5p/NQO1 interplay on metabolic and physiological functions in CRC cells, experiments were conducted in which miR-485-5p mimics or inhibitors, along with vectors for NQO1 modulation, were introduced into the HCT-116 and DLD-1 cell lines. Incorporating the miR-485-5p mimics observably decreased glucose consumption, lactate secretion, and ATP production (Fig. 7A). Furthermore, the upregulation of NQO1 expression mitigated the influence of miR-485-5p on these metabolic processes. Analyses of cell proliferation and migration revealed the suppression of both cellular behaviors upon treatment with miR-485-5p mimics. However, these suppressive effects were reversed on restoring NQO1 levels (Fig. 7B–F). Overall, these findings support the theory that miR-485-5p modulates CRC cell growth and metastatic potential, in part via influencing NQO1 expression and consequently altering energy metabolic pathways.

## Discussion

NQO1 expression varies between neoplastic and normal cells [19] and has garnered attention in tumor detection probe development owing to its distinct expression levels. Clinically, NQO1 is highly overexpressed in many human cancers, including breast [20], liver [21], ovarian [22], and pancreatic [23] cancers, compared with normal tissues. Our study confirmed that NQO1 was frequently upregulated in CRC tissues, which was consistent with previous reports [24]. Increased NQO1 expression was strongly associated with unfavorable outcomes, thereby emphasizing its potential as a promising novel independent prognostic indicator for patients diagnosed with CRC.

Apoptosis is widely recognized as a critical mechanism for preventing tumor development, and a hallmark of tumor cells is their ability to evade apoptosis [25]. Anoikis, a specific form of apoptosis, shares similar molecular mechanisms with conventional apoptosis, exhibits characteristic apoptotic morphological features, and involves the activation of mitochondrial pathways and/or death receptor pathways mediated by caspase family proteins. As the formation of an anoikis-resistant phenotype represents a key initiating step in tumor metastasis, preserving the functional integrity of anoikis is crucial for inhibiting metastatic progression. Based on this premise, we hypothesize that NQO1 plays a potential role in regulating anoikis in CRC cells. In this study, we validated our hypothesis that NQO1 confers resistance to anoikis in CRC cells, thereby promoting CRC metastasis, using an in vitro anoikis model established by culturing cells on poly(2-hydroxyethyl methacrylate)-coated plates.

Cancer cells frequently undergo dysregulated metabolic alterations, with one of the most notable changes being the transition from mitochondrial oxidative phosphorylation to enhanced aerobic glycolysis, commonly known as the Warburg effect [26, 27]. Several studies indicate that tumor cells may enhance aerobic glycolysis and energy production for cell growth via modulating NQO1 expression [28]. NQO1 primarily participates in intracellular redox reactions and plays a key role in regulating the ratios of NAD⁺/NADH and NADP⁺/NADPH. Since glycolysis depends on NAD⁺ as a cofactor to sustain pyruvate production, NQO1’s modulation of the NAD⁺/NADH ratio can indirectly influence glycolytic efficiency [29]. Furthermore, NQO1 may interact with other metabolic regulatory proteins, such as AMPK or mTOR, to regulate cellular energy sensing and metabolic reprogramming, thereby impacting the overall glycolytic activity [30]. Dimri et al. [31] demonstrated that NQO1 stimulated liver cancer cell proliferation via the PI3K/Akt and MAPK/ERK pathways, thereby increasing the expression of key aerobic glycolysis-associated genes. Similarly, Chen et al. [32] reported that NQO1 silencing markedly decreased hexokinase II expression and inhibited cell proliferation by affecting aerobic glycolysis in non-small cell lung cancer cells. Our previous work demonstrated that NQO1 can bind to pyruvate kinase L/R (PKLR), a key regulator of glycolytic reprogramming in breast cancer, thereby activating the AMPK and AKT/mTOR signaling pathways and inducing glycolytic reprogramming [10]. Based on this, we speculate that NQO1 may interact with multiple key glycolytic enzymes, such as lactate dehydrogenase A (LDHA), in CRC, potentially regulating lactate production and tumor metabolism. However, its specific molecular regulatory mechanism requires further experimental verification. These findings suggest that NQO1 regulates CRC cell proliferation and metastasis via mediating aerobic glycolysis both in vivo and in vitro. Rapid glucose catabolism mediated by glycolysis plays a crucial role in sustaining uncontrolled cell growth and promoting tumor metastasis [33]. Glycolysis inhibitors can reverse the promotion of these cellular behaviors by NQO1. However, the specific regulatory mechanisms underlying these processes remain unelucidated, prompting further investigations into the upstream regulation of NQO1.

Multiple studies have identified miR-485-5p as a tumor-suppressive miRNA that inhibits tumor development and progression, and its downregulation is associated with a poor prognosis in various cancer types [34–36]. Chai et al. [15] found a strong correlation between low miR-485-5p levels, tumor volume, clinical stage, and a shorter survival period in patients with CRC. miR-485-5p modulates Bmi-1 acetylation via targeting O-GlcNAc transferase, thereby inhibiting CRC cell proliferation. Additionally, studies by Liu et al. [37] and Wang et al. [38] have shown that miR-485-5p can regulate CRC progression by acting as a sponge for the circular RNAs APLP2 and NOX4, which affect FOXK1 and GKS1B expression. However, the role of miR-485-5p in regulating aerobic glycolysis in CRC remains unclear. Predictive analyses using online databases indicated that NQO1 was a potential target of miR-485-5p. This assertion was substantiated by a luciferase reporter assay, confirming that miR-485-5p targeted the 3′-UTR region of *NQO1*, modulating its expression. Chen et al. [39] identified *NQO1* as a direct miR-485-5p target in lung adenocarcinoma; NQO1 affected its progression via the PI3K/Akt signaling pathway. Our research parallels this discovery, indicating that NQO1 can mitigate the inhibitory influence of miR-485-5p on the aggressive traits of CRC cells. This suggests that the miR-485-5p-mediated suppression of CRC progression is, to some extent, attributable to NQO1 downregulation. In summary, this study established a correlation between high NQO1 expression in CRC tissues and poor patient prognosis. Furthermore, a novel paradigm was delineated, wherein the miR-485-5p/NQO1 axis contributed to CRC progression via promotion of aerobic glycolysis (Fig. 8). These findings demonstrate that the miR-485-5p/NQO1 axis is a pivotal factor in CRC onset and progression and highlight its potential as a crucial molecular marker for early diagnosis, prognostic assessment, and targeted therapy of CRC.

## Electronic supplementary material

Below is the link to the electronic supplementary material.

Supplementary Material 1

## Data Availability

No datasets were generated or analysed during the current study.
