# Supplementary material for: miR-485-5p/NQO1 axis drives colorectal cancer progression by regulating apoptosis and aerobic glycolysis
Source: Cancer Cell Int. 2025 Feb 12;25:41. doi: 10.1186/s12935-025-03672-7 (PMC11823044; doi:10.1186/s12935-025-03672-7)
Supplement: Supplementary file 1 — Supplementary Material 1 [file 12935_2025_3672_MOESM1_ESM.docx]

**Table S1. Primers used in quantitative real time RT-PCR**

| **Primers** | **Sequences** |
| --- | --- |
| GAPDH (forward) | 5’- TCGTGGAAGGACTCATGACC -3’ |
| GAPDH (reverse) | 5’- GGCAGGTTTTTCTAGACGGC -3’ |
| NQO1 (forward) | 5’- GGCAGAAGAGCACTGATCGTA -3’ |
| NQO1 (reverse) | 5’- TGATGGGATTGAAGTTCATGG -3’ |
| miRNA mimic Ncontrol  miRNA inhibitor Ncontrol | UUUGUACUACACAAAAGUACUG  mCmAmGmUmAmCmUmUmUmUmGmUmGmUmAmGmUmAmCmAmAmA |
| hsa-miR-485-5p mimic | AGAGGCUGGCCGUGAUGAAUUC |
| hsa-miR-485-5p inhibitor | mGmAmAmUmUmCmAmUmCmAmCmGmGmCmCmAmGmCmCmUmCmU |
